# The Cortisol Levels, Histology, and Fine Structure of Various Tissues of Fish *Gambusia affinis* (Baird and Girard, 1853) after Exposure to Lead

**DOI:** 10.1155/2023/6649258

**Published:** 2023-11-24

**Authors:** Moh Awaludin Adam, Agoes Soegianto, Yenny Risjani, Carolyn Melissa Payus, R. Gunawan Pratama Yoga, Nina Hermayani Sadi, Evi Susanti, Ach Khumaidi, Ramli Ramli

**Affiliations:** ^1^Faculty of Science and Technology, Universitas Ibrahimy, Situbondo, Indonesia; ^2^Research Center for Marine and Land Bioindustry, National Research and Innovation Agency, Mataram, NTB, Indonesia; ^3^Department of Biology, Faculty of Science and Technology, Universitas Airlangga, Surabaya, Indonesia; ^4^Faculty of Fisheries and Marine Science, University of Brawijaya, Malang, Indonesia; ^5^Faculty of Science and Natural Resources, Universiti Malaysia Sabah, Kota Kinabalu, Sabah, Malaysia; ^6^Research Center for Limnology and Water Resources, National Research and Innovation Agency, Jakarta, Indonesia

## Abstract

**Background:**

Aquatic organisms demonstrate a high vulnerability to mortality when exposed to Pb, even at low concentrations. The objective of this investigation is to ascertain the histopathological alterations and cortisol concentrations in diverse tissues of *Gambusia affinis*, with a specific focus on the eggs and larvae, following exposure to varying concentrations of PbCl_2_.

**Methods:**

Adult specimens of *G. affinis* measuring 5-6 cm in length were obtained from a commercial fish breeding facility. A total of 8 fish with a 1 : 1 ratio of 4 pairs of broodstock were placed in an 8-liter aquarium. Following the adaptation phase, the broodstock underwent a spawning process that lasted for a duration of 7 days. Throughout the spawning process, assessments were conducted on the progression of the abdominal growth of the broodstock. Eggs ready to hatch and *Gambusia* larvae were taken and exposed to 0.1 mg/L PbCl_2_, 1 mg/L PbCl_2_, and control (without PbCl_2_) for 24 hours, with three replications. At the end of the experiment, histopathological analysis was conducted using the hematoxylin Ehrlich-eosin staining method and scanning electron microscopic (SEM) observation. The levels of Pb in gills were determined by employing atomic absorption spectrophotometer. The cortisol concentration in organ samples of fish was determined through the utilization of a cortisol ELISA Kit.

**Results:**

The findings of this investigation demonstrated an important bioaccumulation occurrence of Pb within the gills of *Gambusia* fish that were specifically subjected to 0.1 and 1 mg/L PbCl_2_. The histological structures of eggs and larvae that were subjected to PbCl_2_ exhibited impairment in comparison to the control group. The present study observed a significant elevation in cortisol levels among fish specimens that were subjected to PbCl_2_ exposure.

**Conclusions:**

The findings of this investigation suggest that the occurrence of Pb is linked to a rise in cortisol concentrations in various organs of *G. affinis* larvae. Furthermore, the research indicates that the exposure to Pb has a notable impact on the histological alterations in the eggs and larvae of *Gambusia* fish, implying that they are undergoing stress as a result of the Pb exposure.

## 1. Introduction

Pollution in aquatic environments is a growing problem that needs immediate attention. Typically, increased domestic, agricultural, and industrial activities are responsible for this phenomenon. Heavy metals are regarded as environmental pollutants due to their toxic nature and propensity to accumulate through biological processes [1–3]. Additional characteristics of heavy metals include their propensity to precipitate, their capacity to be assimilated into sedimentary particles, and their ability to remain suspended in aqueous solutions. Metals can be absorbed by fish through their gills and skin, and/or by ingestion through their diet, resulting in their accumulation and potential toxicity [4, 5]. The intensity of bioaccumulation and its toxicity can be influenced by various environmental factors such as temperature, oxygen levels, pH, and water hardness.

Lead (Pb) is classified as a hazardous heavy metal that possesses a high propensity for absorption and accumulation [6–8]. Pb is an inherent constituent of the environment and is typically present in trace quantities within the soil, flora, and aqueous systems. Pb has been detected in aquatic environments, and its concentration has been observed to rise as a result of human activities such as producing batteries, painting, and cement manufacturing [9–11]. The results of investigations conducted by Dimari et al. [12], Hasan et al. [13], and Odey et al. [14] in various lakes and streams have revealed the presence of elevated concentrations of lead (Pb) in the water, sediment, and aquatic organisms, including fish. The rising amount of Pb-containing waste in aquatic environments leads to an increase in Pb contamination. This substance has a propensity to form diverse chemical bonds with oxygen and sulfur groups found in protein molecules. The increased ability of these elements to form stable complexes increases the bioavailability of lead within proteins. Hypocalcemia may result from the presence of lead in biota, such as fish. The high affinity of Pb for Ca^2+^ ATPase, Na^+^/Ca^2+^ substitution, and Na^+^/K^+^ ATPase results in the inhibition of the ionocyte lateral transport mechanism in the gill epithelium [1]. These effects can disrupt electrochemical gradients and the regulation of ions. Due to bioaccumulation, exposure to Pb can be lethal for aquatic organisms, even at low concentrations [12–14]. Pb can disrupt antioxidant equilibrium, cause oxidative stress disorders, and change behavior [15–18]. In vivo experiments on the effects of Pb exposure in fish have examined the augmentation of antioxidant responses by inducing reactive oxygen stress (ROS), the toxic effects on membrane structure and function due to Pb's high affinity for red blood cells, and the changes in fish immune systems [5, 19–21]. *Gambusia affinis*, commonly known as mosquito fish, is one of the preferred fish species utilized as a biomonitoring tool in contaminated water bodies.

Several studies [22–25] have previously investigated the levels of stress in fish and biota aquatic exposed to heavy metals. Plasma cortisol levels were elevated in two-month-old *Oreochromis mossambicus* fish exposed to Cu but not in fish exposed to the combination of Cu and Cd [26]. Adult *O. mossambicus* exposed to Cd for 2, 4, or 14 days showed a substantial increase in plasma cortisol levels [27]. During the exposure phase, the level of cortisol produced by adult *Oreochromis* sp. treated with Pb or As was less than that of the control group [22]. Cu-exposed rainbow trout (*Oncorhynchus mykiss*) did not exhibit a stress response to cortisol [26, 28, 29], whereas Cr exposure induced deleterious effects on the stress indicator cortisol [30–32] observed that zebrafish (*Danio rerio*) experienced stress in response to a sudden change in temperature. Meanwhile, research on the effects of Pb exposure [22, 33] indicates that Pb interferes with endocrine function by altering the pattern of synthesis and metabolism of cortisol and growth hormone (GH) in adult *Orechromis* sp. and *Cyrpinus carpio*. Other studies [26, 27, 34] found that the gills of Atlantic salmon (*Salmo salar*) were unaffected by Cd exposure via fish diet. Limited research has been undertaken concerning the impact of Pb on the early stages of fish development, particularly in relation to fish eggs and larvae. The objective of this study was to evaluate cortisol levels in different organs of fish larvae of *Gambusia affinis*. The purpose was to evaluate the stress levels experienced by the larvae following exposure to Pb. Furthermore, histological and fine structural examinations were conducted on these essential organs.

## 2. Materials and Methods

### 2.1. Adaptation of Fish to Laboratory Conditions

The mosquito fish (*Gambusia affinis*) broodstocks, measuring 5-6 cm in length, were obtained from the Punten Fish Seed Center (PFSC) located in Batu, East Java, Indonesia. The PFSC water quality consisted of the following characteristics: temperature of 23–26°C, pH of 6.5–7, and lead concentration of less than 0.002 mg/L. The mosquito fish broodstock were reared in 60-liter tanks within the laboratory setting. During the adaptation period, *Gambusia* broodstock were given food with fish pellets twice daily for a duration of 14 days. Additionally, daily water changes were conducted, amounting to 20% of the total water volume. Each aquarium was stocked with eight broodstock fish, maintaining a 1 : 1 ratio of four pairs of broodstock in an 8-liter tank. Throughout the acclimatization and testing periods, the temperature, pH, and dissolved oxygen levels were continuously monitored in accordance with the guidelines established by Berlinsky et al. [35]. The temperatures ranged from 27 to 29°C, the pH was between 6 and 7, and there was more than 4 mg/L of dissolved oxygen.

### 2.2. Spawning and Pb Exposure

Following the adaptation period, the broodstock undergo spawning in specialized tanks for approximately seven days. Throughout the spawning process, the progression of the female parent's abdomen development was monitored. Following a period of 7 days, a surgical procedure was conducted on the distended abdomen of the female parent. Before the surgical procedure, the fish specimens were subjected to anesthesia using 0.2 mL of clove oil [36]. Subsequently, the surgical intervention was carried out, and the eggs and larvae were cautiously extracted. After that, eggs that were in a state of readiness to hatch and larvae of *Gambusia* were subjected to varying concentrations of PbCl_2_ for a duration of 24 hours.

The concentrations utilized in the present investigation were selected based on the findings of Al-Kshab and Yehya [37], who documented that adult *Gambusia* fish exposed to PbCl_2_ exhibited LC_50_ 24-h and 96-h values of 59 and 50 mg/L, respectively. Due to the use of *Gambusia* fish eggs and larvae as research subjects, the concentrations of PbCl_2_ (Merck, Darmstadt, Germany) used in this study were 0.1 mg/L (A) and 1 mg/L (B), while the control group contained no PbCl_2_. Ten eggs and ten larvae were separately introduced into separate 1-liter glass jars, which were filled with 500 ml and 750 ml of experimental media, respectively. The study was conducted using three replicates. The experiment lasted for 24 h. The larvae were then euthanized by rapid freezing and followed by rapid freezing in liquid nitrogen to stop any enzymatic activity [38]. The eggs were quickly processed for scanning electron microscopy examination. The present study was carried out in accordance with the Institutional Animal Care guidelines and procedures of Brawijaya University (number 958-KEP-UB).

### 2.3. Pb Analysis

In order to determine the levels of metal present in the water used for metal treatment and gills of *Gambusia* fish, measurements were taken. A total of 200 mL water sample was put into a 250 mL beaker containing 2 mL of 1% ammonium pyrollidine dithiocarbamate (APDC), pH was set to 4, and heated to boiling. After cooling down to room temperature, place in Erlenmeyer flask, and add 7 mL of methyl isobutyl ketone (MIBK), then shake with a shaker for 20 minutes. The solution was put into a separatory funnel and left for 20 minutes. Organic layer (top) was taken and placed it in the Erlenmeyer flask. For re-extraction, 5 mL of 4N HNO_3_ was pipetted and added and then stirred for 20 minutes. Later, the mixture was put into a separatory funnel until the boundaries were found (±20 minutes). The bottom layer (acid layer) was taken and analyzed with Atomic Absorption Spectrophotometer (AAS) Shimadzu AA-6800 [39]. The similar procedure was applied for standard solutions of the metal being analyzed. The purpose of preconcentration of the sample by the solvent extraction method is to separate the metal ions which are determined in the measurement with the AAS [40]. CASS-6, a certified reference material (CRM) of seawater provided by the National Research Council, Canada (NRCC), was used to check the accuracy of the measurements and showed good recoveries (91%).

Before measuring the level of Pb in gills of *Gambusia* fish, the fish gill specimens were subjected to oven-drying at a temperature range of 90–100°C for a duration of 12 hours. Subsequently, the dried samples were pulverized and weighed, resulting in 0.5 g. To establish a control, introduce 0.25 ml of standard solution containing 1 mg/L of Pb to the sample prior to its placement in the ashing furnace. Subsequently, subject the sample to steam with a hot plate until it is completely dry. Introduce the sample into the ashing furnace. The recommended process is to gradually raise the temperature by 100°C every 30 minutes until it reaches 450°C, and then maintain it at that level for a duration of 18 hours. The process of measuring Pb in water was applied to the examination of fish gills. The analytical performance metrics such as accuracy and precision were assessed through the measurement of those of the dogfish muscle reference material (DORM-4) that was supplied by the NRCC. The reference material of the NRCC was subjected to identical measurement techniques as those used for the samples. The metal recovery acquired from the analysis of certified reference material (DORM-4) was 92%.

### 2.4. Histopathological Analysis

The preparation was conducted subsequent to the conclusion of the PbCl_2_ exposure duration. The procedure for preparation uses conventional techniques as described in [41]. Initially, the fish eggs, larvae, and gills were promptly immersed in a 10% phosphate-buffered formalin (NBF) fixative and allowed to incubate for a duration of 24 hours. Afterwards, they were rinsed with 70% alcohol. Following fixation, the tissue undergoes processing with paraffin, which endeavors to preserve the integrity of cells and tissues, thereby facilitating the embedding process. Subsequent to the tissue embedding procedure, the resultant blocks were subjected to tissue sectioning utilizing a microtome with an incision thickness ranging from 3 to 5 *µ*m. The stretched cut ribbon underwent a process of being immersed in a water bath maintained at a constant temperature of 40°C. The objective lens is utilized to immerse the tape in a water bath and subsequently subject it to a one-hour drying period. Subsequently, the tissue slides were subjected to a clearing process using a xylol solution. Following this, the slides were allowed to dry and subsequently stained using the hematoxylin Ehrlich-eosin staining method. Finally, the slides were mounted with entellan and covered with a cover glass. The study involved the utilization of an Olympus CX33 microscope and the EPview 1.2 application (Build 19853) for observation purposes.

### 2.5. Scanning Electron Microscopic (SEM) Observation of Fish Eggs and Larvae

The preparatory phases preceding SEM observation are crucial in achieving optimal image quality outcomes and facilitating accurate analysis. It is imperative that any treatments administered during the sample preparation process do not alter the inherent structure of the sample, in order to ensure that the observations obtained through SEM accurately reflect the original structure of the sample. The initial stages of preparing biological samples for observation via SEM involve several crucial steps, namely, orienting the sample to the desired position, fixing it in place, dehydrating it, drying it, and finally, applying a conductive coating. A modification of this method has been proposed by the authors in [42]. The fixation stage is undertaken to maintain the original structure of the sample and prevent any potential collapse or fragility. The fixation process generally comprises two discrete phases. In the first phase, glutaraldehyde and cacodylate buffer were employed, followed by the application of osmium tetroxide in the buffer during the subsequent phase.

The process of dehydration involved the removal of water molecules from the sample. The process of dehydration was executed through submersion in alcohol, wherein the concentration level was incrementally augmented until it attained a maximum of 100%. The process of drying was executed through the utilization of critical point drying (CPD) as described in [43]. Additionally, hexamethyldisilazane was used to eliminate the liquid component of the sample while avoiding any potential deflation of the sample. The process of applying conductive coatings can be achieved through the utilization of a sputtering apparatus in conjunction with gold as conductive material. The study involved the examination of various types of samples, including conductive samples and biological samples that were both prepared and unprepared. The observation was conducted using a specialized mode of observation known as variable pressure scanning electron microscopy (VP-SEM). The Hitachi SU3500 scanning electron microscope was utilized for observations at the Biology Research Center, facilitated through the Science E-Service Application (ELSA) provided by the National Research and Innovation Agency (BRIN).

### 2.6. Cortisol Levels Measurement

The present study used a standardized protocol [44] utilizing a Cortisol ELISA Kit (ab108665), United States, to measure the concentration of cortisol in various organ samples of fish, including gills, gonads, meat, liver, eyes, and fins. To prevent the impact of washing, it is recommended to increase the volume of washing solution from 300 *µ*L to 350 *µ*L and perform washing steps three to five. Duplicate all assays for standards, controls, and samples. Ensure that all reagents, working standards, and samples are prepared according to the ELISA Kit instructions. Eliminate surplus microplate strips from the plate frame, restore them to the foil pouch that encloses the desiccant pack, seal again, and put them back into storage at a temperature of 4°C. Subsequently, 20 *µ*L of standard, control, or sample was introduced into their corresponding wells, followed by the addition of 200 *µ*L of cortisol-HRP conjugate to each well. A blank well was left to accommodate the substrate material. The wells were covered with the foil provided in the kit. The sample was subjected to incubation for a duration of one hour at a temperature of 37°C. Upon completion of the incubation process, the foil was removed and the contents of the wells were aspirated. Subsequently, each well was washed thrice with 300 *µ*L of diluted washing solution, which prevented the occurrence of overflow in the reaction wells. It is recommended that the duration of the soak time between consecutive wash cycles should exceed 5 seconds. Subsequently, the residual fluid was meticulously eliminated by gently tapping strips onto tissue paper prior to proceeding with the subsequent stage. 100 microliters of TMB substrate solution was introduced into all wells. The sample was subjected to incubation for precisely 15 minutes under ambient conditions in the absence of light. Subsequently, 100 *µ*L of stop solution was introduced into each well in a consistent manner and sequence as that of the TMB substrate solution, which gently agitated the microplate. The emergence of a blue hue during the incubation period results in a subsequent transformation into a yellow hue. The absorbance of the sample was measured at a wavelength of 450 nm, using an automatic ELISA Microplate Reader Epoch (Biotek), within a timeframe of 5 minutes following the addition of the stop solution.

### 2.7. Data Analysis

A descriptive analysis was conducted on the histochemistry and SEM observations of a number of different organs. The bioaccumulation of lead in gills and cortisol levels in various organs were statistically analyzed using analysis of variance (ANOVA). Subsequently, the Tukey HSD post hoc test at a significance level of 5% should be conducted to determine the differences between each treatment. The statistical analysis of the level of cortisol found in various organs, as well as the levels of lead found in the gills and the water used as treatment, are reported in the supplementary data.

## 3. Results

The results of exposure to Pb concentrations in this study indicated the presence of a bioaccumulation process in the gills of the model fish, *Gambusia*.  Table 1 presents a summary of the lead accumulation in the gills as well as the lead concentration in the medium. There were statistically significant differences (*p* < 0.005) between each treatment and media.

### 3.1. Histological Observations

**Figure 1:**
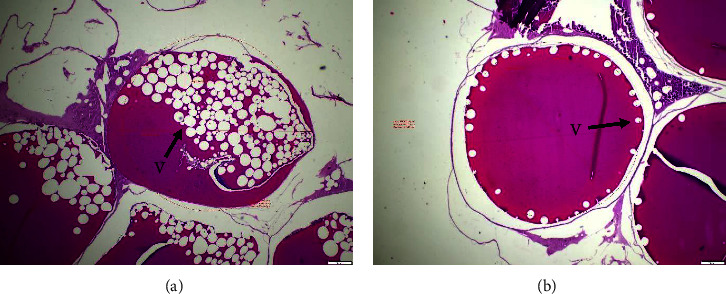
Observation of egg structure by HE staining. (a) Visible structure of fish eggs exposed to PbCl_2_ and (b) visible structure of control fish eggs without exposure of PbCl_2_. V = vacuolla and bar size = 200 *µ*m.

**Figure 2:**
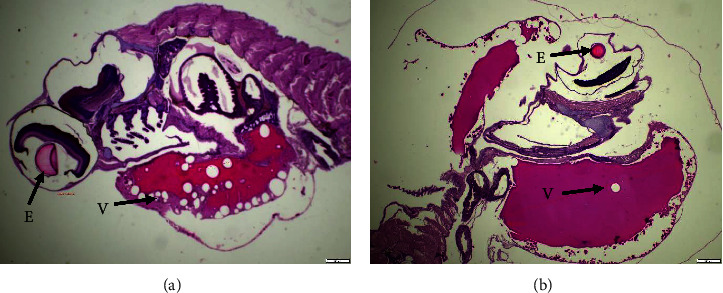
Observation of the structure of *Gambusia* fish larvae with HE staining. (a) Fish larvae exposed to PbCl_2_ and (b) control fish larvae without exposure of PbCl_2_. E = eye, V = vacuolla, and bar size = 200 *µ*m.

**Figure 3:**
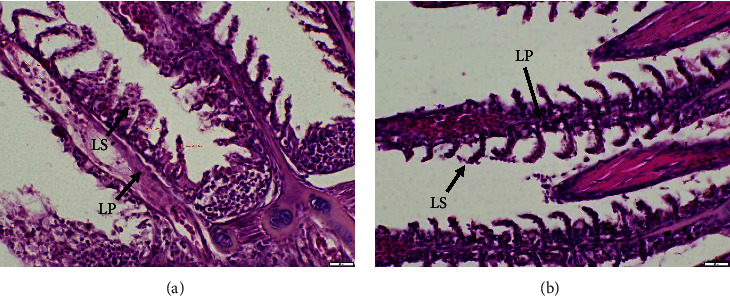
Observation of gill structure of *Gambusia* fish with HE staining. (a) Swelling gill structure of fish exposed to Pb and (b) visible gill structure of control fish without exposure. LS = secondary lemella, LP = primary lamellae, and bar size = 200 *µ*m.

Histological impairment was observed in fish egg, larvae, and gills. A discernible alteration can be shown in the results of the analysis conducted on fish eggs (Figure 1), the assessment carried out on fish larvae (Figure 2), and the examination performed on fish gills (Figure 3).

Figure 1 shows the progression of egg cell division, specifically during phases I-III of vitellogenesis, which can be identified by the emergence of vacuoles. PbCl_2_-exposed egg cells (A) exhibited irregularly shaped vacuoles, resulting in a significant difference between treatments A and B. The proper positioning of the vacuole on the periphery of the egg is crucial in the process of vitellogenesis. Exposure to PbCl_2_ results in a formation of vacuoles, which remain localized in the central region of the egg. In contrast to the control group (B), it is evident that the development characterized by typical vacuoles is situated at the periphery of the egg.

A discernible variation between the Pb exposure treatment (A) and the control (B) is evident in the larval development process (Figure 2). The exposure of fish larvae to Pb resulted in an incomplete development of the eyes, characterized by the presence of imperfect spheres. Similar to the progression of yolk development, it was expected that the vacuole within the egg cell would undergo nuclear transformation. However, it has been observed that vacuoles persist within the yolk of the larvae. In contrast to the control fish larvae, it appears that the morphogenesis of ocular cells exhibits a perfectly spherical shape. Similarly, during the process of yolk development, the presence of vacuoles is limited due to the formation of a nucleus.

Differences were observed in the gills of fish larvae that were exposed to PbCl_2_ (A) in comparison to the control group (B). The findings from the examination of fish larval gills that were subjected to PbCl_2_ (A) exposure indicate that both the primary lamellae (LP) and secondary lamellae (LS) experienced a process of swelling. In contrast, the gills of the experimental fish (A) exhibited no alteration in the LP and LS.

### 3.2. SEM Observation

**Figure 4:**
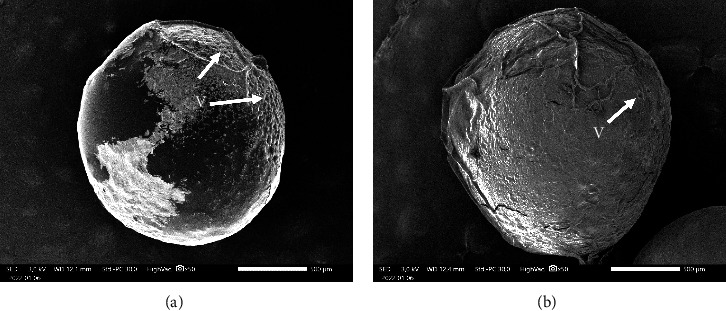
Observation of egg structure with SEM observation (a) on the surface of the eggs of fish exposed to PbCl_2_, the vacuolization structure is evident and (b) visible structure of control fish eggs without exposure of PbCl_2_. V = vacuola and bar size = 500 *µ*m.

**Figure 5:**
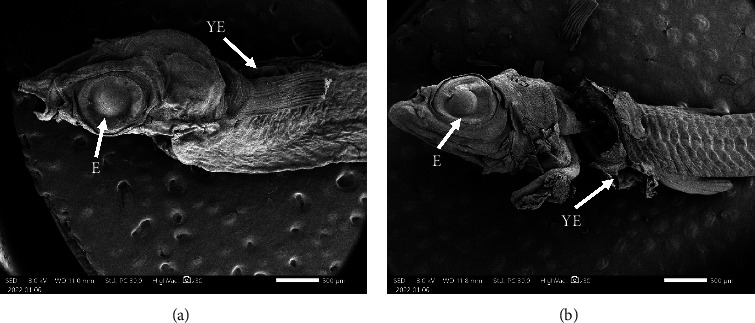
Observation of the structure of fish larvae with SEM observations. (a) The development of ocular structures with nonuniform circularity structure of fish larvae exposed to PbCl_2_ and (b) optimal spherical morphology of ocular structures of control fish larvae without exposure of PbCl2. E = eye, YE = yellow egg, and bar size = 500 *µ*m.

**Figure 6:**
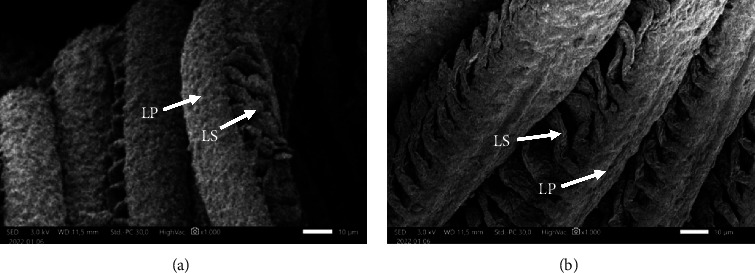
Observation of fish gill structure with SEM observation. (a) Swelling gill structure of fish exposed to PbCl_2_ and (b) normal gill structure of control fish without exposure of PbCl_2._ Bar size = 10 *µ*m, LS = secondary lamellae, and LP = primary lamellae.

The SEM observations provided additional elucidation on the structural alterations resulting from PbCl_2_ exposure in fish eggs, as depicted in Figure 4. The observations of fish larvae and fish gills are presented in Figures 5 and 6, respectively.

The findings obtained from histological analysis using HE staining are consistent with those derived from scanning electron microscopy, indicating that egg cells are undergoing vacuolization. A noticeable contrast was observed between treatments A and B. Specifically, the egg cells that were subjected to PbCl_2_ (A) exhibited an irregular shape in their vacuoles. The localization of vitellogenesis vacuoles in the egg should be peripheral during its development. Exposure to PbCl_2_ (A) results in the accumulation of vacuoles, which remain localized on a single side of the egg. In contrast to the control group (B), it appears that the development of egg cells is normal with no vacuole accumulation.

Differences were observed between the PbCl_2_ treatment (A) and the control (B) in terms of larval development, as indicated by the results of SEM observations. The exposure of fish larvae to PbCl_2_ resulted in the development of ocular structures with nonuniform circularity. They have a tendency to exhibit an oval shape and an asymmetrical enlargement on one of their sides in contrast to the control group, who exhibited ocular development characterized by a more optimal spherical morphology. However, the development of the egg yolk appears to differ from the results obtained through HE histology, which clearly demonstrate variations in the yolk.

SEM observation of the gills of fish larvae revealed distinct morphological variations between the larvae that were subjected to PbCl_2_ (A) and the control group (B). The gills of fish larvae A exhibited swelling in LP and LS, similar to the histological distinctions observed through HE staining. Conversely, the gills of fish B did not display any alterations in LP and LS. The gills of the control fish exhibited typical developmental patterns.

### 3.3. Measurement of Cortisol Levels

**Figure 7:**
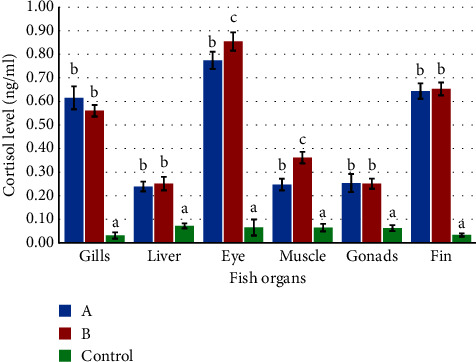
Cortisol levels in fish organs exposed to lead. *A* = 0.1 m/L PbCl_2_ and *B* = 1 mg/L PbCl_2_. Lowercase letters denote statistically significant differences (*a* < *b* < *c*, *p* < 0.05). The sample size is *N* = 5, with each sample comprising a composite of two individuals.

According to the cortisol biomarker analysis, it was determined that the stress level of all fish organs exposed to PbCl_2_ was significantly higher than that of the control group. The cortisol levels of fish organs in treatments A and B were found to be nearly equivalent (Figure 7).

## 4. Discussion

Metals are released into the aquatic environment and subsequently absorbed by fish through their gills [45]. The metals subsequently accumulate in the fish organs after flowing via the circulatory system, whilst certain metals are eliminated via the renal and branchial excretory systems [46–49]. Pb is considered to be one of the most accumulative metals due to its capacity to bind with oxygen and sulfur atoms in complex proteins. As a result, it is classified as a toxic metal [50–52].

Exposure to Pb has the potential to cause damage to reproductive organs. Research has demonstrated that the presence of pollutants can impede the growth and maturation of reproductive cells in zebrafish (*Danio rerio*), with a particular impact on primordial germ cells [53–55]. It has been documented that the exposure to pesticides of the organophosphate type can impede the production of estrogen hormone, thereby disrupting the vitellogenesis process [56–58]. Comparable results were also found in this study, as shown by the HE staining shown in Figure 1 and the results of the SEM observation shown in Figure 4, respectively. The presented figures demonstrate an interruption in the vitellogenesis process, characterized by anomalous vacuole formation, which can be attributed to the exposure to Pb. The findings of Ansari et al. [59], Aleström et al. [60], and Parsons [61] indicate that interference with vitellogenesis leads to a reduction in the size of fish eggs and larvae. The exposure to Pb has a persistent impact on the eggs of fish, which subsequently translates into the morphology and physiology of the resulting fish larvae. The results of this study, as demonstrated by the HE staining in Figure 2 and the SEM images in Figure 5, indicate the presence of malformations in fish larvae. Exposure to Pb can result in enlarged eyes and noncircular eyelids. The zebrafish (*Danio rerio*) larvae exposed to Hg exhibit various malformations and abnormalities, including pericardial edema, spinal curvature, and fin edema [47, 62, 63]. The exposure of fish to Pb has been found to interfere with the spawning process and result in an increase in morphological abnormalities in fish sperm [45, 64, 65]. Additionally, Pb that passively diffuses into the egg yolk has been shown to inhibit the activity of embryo growth enzymes [57, 66, 67]. Moreover, it has been demonstrated that Pb exposure can impede the embryogenesis process in goldfish (*Cyprinus carpio*), resulting in blastula defects, spinal curvature, and heart defects [60, 68].

Even though the organism is able to withstand the initial impact of the pollutant, prolonged exposure causes its self-defense mechanisms to become less effective [8, 69]. The ability of the body to tolerate pollution, even at low quantities, can decline with age. Furthermore, metal toxicity is affected by the organism's sensitivity to the metal, environmental factors, and the metal's excretion and retention rates [51, 52, 58]. The accumulation of metals in fish tissues depends on several factors, including the concentration and duration of exposure, as well as additional variables such as temperature, age, interaction with other metals, water chemistry, and metabolic activity of the fish [3, 14, 70]. Lead is not considered an essential element for biological processes. Human activities may contribute to the release of excessive levels of lead into nearby water sources [1, 46].

The toxicity of Pb is evident even at low concentrations, and its involvement in biochemical processes remains unclear [14, 37]. It has been observed that fish are capable of accumulating heavy metals in their tissues at a higher concentration than the surrounding environment. This is achieved through the absorption processes that occur along the surface of their gills and kidneys, liver, and walls of the intestinal tract [8, 12, 71, 72]. This study demonstrated the presence of lead in the gill tissue of *G. affinis.* The findings indicate that there was a discernible mechanism of Pb uptake in the gills, which resulted in the enlargement of both the primary and secondary lamellae. According to Usman et al. [1], the impairment of gill lamellae may lead to disturbances in ion regulation within the gills, which can ultimately affect the homeostatic mechanisms responsible for maintaining ion levels. The gills serve as a crucial point of entry for heavy metals and are typically the primary organ affected by metal exposure in fish [3, 73–75]. According to Mahboob et al. [76], there was a higher concentration of lead detected in the gills of Nile tilapia (*Oreochromis niloticus*) compared to the kidneys, liver, and muscles. The high accumulation of metals in the gills has been attributed to the formation of metal-mucus complexes [77–79]. The concentration of metals in the gills of fish serves as an indicator of the corresponding metal levels found in the surrounding aquatic environment [80–82]. Thus, fish gills are frequently suggested as an environmental indicator organ for fish.

In addition to organ damage, exposure to heavy metals can also result in elevated stress levels [47, 59]. The activation of the hypothalamus-pituitary-adrenal (HPA) axis is a commonly observed mechanism for effectively responding to stressors [62, 83, 84]. Cortisol is the primary corticosteroid synthesized by the hypothalamic-pituitary-adrenal (HPA) axis in the majority of mammals and fish [83, 85]. Cortisol secretion in response to stress is influenced by a number of stimuli, including toxin exposure, transport, and shock. Corticosteroids primarily induce the mobilization of energy reserves [86] via glycolysis and gluconeogenesis, thereby facilitating the achievement of the organism's energetic demands in response to the particular conditions [87]. The results of the study demonstrate an increase in cortisol levels as a result of Pb exposure. Fish gills, eyes, fins, liver, gonads, and muscle have significantly different cortisol concentration increases, as shown in Figure 7. Cortisol concentrations in these organs correlate positively with Pb concentrations in the surrounding environment. According to studies conducted by Tang et al. [22] and Ramesh et al. [33], the impact of Pb exposure has been found to interfere with endocrine function by altering the synthesis and metabolism of cortisol and growth hormone (GH). The consequences of the mentioned exposure may have negative effects on the health of fish. Previous research conducted by Pelgrom et al. [26], Pratap and Bonga [27], and Dang et al. [34] has suggested that the ingestion of Cd by Atlantic salmon via their dietary intake did not yield any observable effects on their gills. Interestingly, it is possible that the accumulation of Cd in the chloride cells of the gills may have led to an upregulation of metallothionein (MT) expression, which plays a crucial role in minimizing the toxic effects of heavy metals. Several studies have reported that rainbow trout did not exhibit a stress response to cortisol when exposed to Cu [26, 28, 29]. Conversely, exposure to Cr induced toxic effects on the stress indicator cortisol in rainbow trout [30, 31]. Additionally, Deniro and Yusnaini [32] observed stress in zebrafish (*Danio rerio*) due to temperature shock. According to several studies, exposure to temperature shock in zebrafish results in physiological alterations in vertebrate animals, leading to a state of exhaustion as a stress response [84, 88, 89]. Deniro and Yusnaini [32] proposed that zebrafish exhibited behavioral responses to stress induced by temperature shock. In response to environmental stimuli, sudden shifts in the equilibrium of fish behavior cause stress. Cortisol, an indicator of prolonged physiological stress, has been linked to the manifestation of stressful behavior [60, 83, 90]. This phenomenon can be observed through the behavior of fish when exposed to stressors; they exhibit sudden bouts of high-velocity swimming and move toward the inlet. It is postulated that under such circumstances, the piscine species exhibit elevated levels of cortisol. According to Barcellos et al. [83, 91], the experimental study has demonstrated that zebrafish exposed to temperature shock prefer the lower area to the upper area, suggesting that cortisol is involved in stress-related behavior. Cortisol in fish is synthesized by inter-renal cells and is found in low concentrations throughout the organism. According to Gris [86], the substance is eliminated in the renal pelvis and subsequently introduced into the bloodstream. Unbound cortisol in plasma is regarded as the sole form that is physiologically active [22, 27]. While the presence of cortisol binding globulin (CBG) has not been identified in fish, various molecules that have an equivalent effect of reducing cortisol bioavailability have been documented [92]. The plasma of salmon is estimated to bind 30–55% of cortisol, with a notably higher percentage observed in females (45%) compared to males (37%). The precise function and characteristics of these molecules that facilitate binding remain unclear [93, 94]. The above discussion is really clear and provides an understanding of the magnitude of the effect of Pb exposure on histopathological changes and stress responses in embryonic and larval *Gambusia* fish.

## 5. Conclusion

To the best of our current knowledge, this study represents the first examination of the impact of lead exposure on fish eggs and larvae. Specifically, it evaluates the impact of lead on cortisol levels within different organs of fish larvae, as well as the consequences of lead exposure on alterations in the morphological structure of fish eggs and gills. According to the findings of this study, the presence of Pb causes an increase in cortisol levels in a number of *G. affinis* larval organs. In addition, the study found that Pb exposure has a significant effect on the histological alterations of *Gambusia* fish eggs and larvae, indicating that the fish are experiencing stress due to Pb exposure. Since Pb is extremely toxic to eggs and larvae of fish, preventing Pb from contaminating aquatic environments is regarded as one of the most significant actions to be taken. In order to prevent contamination of aquatic environments, it is crucial to take immediate action to ensure that future pollution and discharges of Pb in aquatic environments are minimized to the greatest extent possible.

## Figures and Tables

**Table 1:** The average concentration of lead (Pb) in water and the gills of *Gambusia affinis*.

Treatment	Lead in water^*∗*^ (mg/L)	Lead in fish gills^*∗∗*^(mg/kg)
A	0.012 ± 0.001^b^	0.023 ± 0.002^b^
B	0.020 ± 0.001^c^	0.030 ± 0.001^c^
Control	0.005 ± 0.003^a^	0.008 ± 0.001^a^

Lowercase letters indicate statistically significant differences (a<b < c, *p* < 0.05), ^*∗*^measured level of Pb in water (each data from 3 measurements), *A* = 0.1 mg/L PbCl_2_, *B* = 1 mg/L PbCl_2_, Control = without Pb, ^*∗∗*^the sample size is *N* = 5, with each treatment comprising a composite of two individuals.

## Data Availability

The data used to support the findings of this research are available from the corresponding author upon request.

## Supplementary Materials

Supplementary Materials Table 1. Cortisol levels (ng/ml) in fish samples exposed to lead. Table 2. Concentration of Pb (mg/kg) in gills of *Gambusia affinis*. Table 3. Concentration of Pb (mg/L) in media (water).
