# Supplementary material for: The Cortisol Levels, Histology, and Fine Structure of Various Tissues of Fish Gambusia affinis (Baird and Girard, 1853) after Exposure to Lead
Source: Scientifica (Cairo). 2023 Nov 24;2023:6649258. doi: 10.1155/2023/6649258 (PMC10691898; doi:10.1155/2023/6649258)
Supplement: Supplementary Materials — Table 1. Cortisol levels (ng/ml) in fish samples exposed to lead. Table 2. Concentration of Pb (mg/kg) in gills of Gambusia affinis. Table 3. Concentration of Pb (mg/L) in media (water). [file 6649258.f1.docx]

**Supplementary Data**

**Table 1.** Cortisol levels (ng/ml) in fish samples exposed to lead.

| Treatment | Gill | Liver | Eye | Muscle | Gonads | Fin |
| --- | --- | --- | --- | --- | --- | --- |
| 0.1 mg/L PbCl_2_  (A) | 0.60 | 0.23 | 0.80 | 0.24 | 0.23 | 0.65 |
|  | 0.67 | 0.27 | 0.77 | 0.26 | 0.26 | 0.66 |
|  | 0.56 | 0.25 | 0.73 | 0.22 | 0.20 | 0.60 |
|  | 0.58 | 0.21 | 0.75 | 0.28 | 0.27 | 0.62 |
|  | 0.66 | 0.24 | 0.82 | 0.23 | 0.30 | 0.68 |
| 1 mg/L PbCl_2_  (B) | 0.57 | 0.23 | 0.87 | 0.38 | 0.23 | 0.61 |
|  | 0.57 | 0.27 | 0.86 | 0.37 | 0.27 | 0.67 |
|  | 0.53 | 0.22 | 0.83 | 0.33 | 0.25 | 0.64 |
|  | 0.59 | 0.24 | 0.80 | 0.34 | 0.27 | 0.67 |
|  | 0.54 | 0.29 | 0.90 | 0.38 | 0.26 | 0.66 |
| Control (Without PbCl_2_) | 0.03 | 0.07 | 0.03 | 0.05 | 0.05 | 0.02 |
|  | 0.02 | 0.09 | 0.10 | 0.09 | 0.07 | 0.03 |
|  | 0.02 | 0.06 | 0.06 | 0.06 | 0.05 | 0.04 |
|  | 0.05 | 0.08 | 0.10 | 0.07 | 0.08 | 0.03 |
|  | 0.04 | 0.07 | 0.04 | 0.05 | 0.06 | 0.04 |

**Statistical test** **of cortisol levels in different organs**

**Normality test**

| **Tests of Normality** | | | | | | | | |
| --- | --- | --- | --- | --- | --- | --- | --- | --- |
| Treatment | | Kolmogorov-Smirnov^a^ | | | Shapiro-Wilk | | |  |
|  |  | Statistic | df | Sig. | Statistic | df | Sig. |  |
| Gill | Control | 0.227 | 5 | .200^*^ | 0.897 | 5 | 0.395 |  |
|  | A | 0.258 | 5 | .200^*^ | 0.925 | 5 | 0.563 |  |
|  | B | 0.221 | 5 | .200^*^ | 0.902 | 5 | 0.421 |  |
| Liver | Control | 0.127 | 5 | .200^*^ | 0.999 | 5 | 1.000 |  |
|  | A | 0.234 | 5 | .200^*^ | 0.928 | 5 | 0.585 |  |
|  | B | 0.237 | 5 | .200^*^ | 0.961 | 5 | 0.814 |  |
| Eye | Control | 0.162 | 5 | .200^*^ | 0.971 | 5 | 0.884 |  |
|  | A | 0.183 | 5 | .200^*^ | 0.985 | 5 | 0.961 |  |
|  | B | 0.250 | 5 | .200^*^ | 0.862 | 5 | 0.234 |  |
| Muscle | Control | 0.198 | 5 | .200^*^ | 0.957 | 5 | 0.787 |  |
|  | A | 0.265 | 5 | .200^*^ | 0.836 | 5 | 0.154 |  |
|  | B | 0.201 | 5 | .200^*^ | 0.881 | 5 | 0.314 |  |
| Gonad | Control | 0.183 | 5 | .200^*^ | 0.985 | 5 | 0.961 |  |
|  | A | 0.201 | 5 | .200^*^ | 0.881 | 5 | 0.314 |  |
|  | B | 0.221 | 5 | .200^*^ | 0.902 | 5 | 0.421 |  |
| Fin | Control | 0.199 | 5 | .200^*^ | 0.967 | 5 | 0.858 |  |
|  | A | 0.253 | 5 | .200^*^ | 0.854 | 5 | 0.207 |  |
|  | B | 0.231 | 5 | .200^*^ | 0.881 | 5 | 0.314 |  |
| *. This is a lower bound of the true significance. | | | | | | | | |
| a. Lilliefors Significance Correction | | | | | | | | |

**ANOVA**

| **ANOVA** | | | | | | |
| --- | --- | --- | --- | --- | --- | --- |
|  | | Sum of Squares | df | Mean Square | F | Sig. |
| Gill | Between Groups | 1.034 | 2 | 0.517 | 492.400 | 0.000 |
|  | Within Groups | 0.013 | 12 | 0.001 |  |  |
|  | Total | 1.047 | 14 |  |  |  |
| Liver | Between Groups | 0.098 | 2 | 0.049 | 99.041 | 0.000 |
|  | Within Groups | 0.006 | 12 | 0.000 |  |  |
|  | Total | 0.104 | 14 |  |  |  |
| Eye | Between Groups | 1.875 | 2 | 0.938 | 724.964 | 0.000 |
|  | Within Groups | 0.016 | 12 | 0.001 |  |  |
|  | Total | 1.891 | 14 |  |  |  |
| Muscle | Between Groups | 0.223 | 2 | 0.111 | 237.121 | 0.000 |
|  | Within Groups | 0.006 | 12 | 0.000 |  |  |
|  | Total | 0.229 | 14 |  |  |  |
| Gonad | Between Groups | 0.123 | 2 | 0.061 | 96.031 | 0.000 |
|  | Within Groups | 0.008 | 12 | 0.001 |  |  |
|  | Total | 0.131 | 14 |  |  |  |
| Fin | Between Groups | 1.257 | 2 | 0.628 | 1083.460 | 0.000 |
|  | Within Groups | 0.007 | 12 | 0.001 |  |  |
|  | Total | 1.264 | 14 |  |  |  |

**Tukey HSD test**

| **Gills** | | | | | | |  | |  |
| --- | --- | --- | --- | --- | --- | --- | --- | --- | --- |
| Treatment | | N | Subset for alpha = 0.05 | | | |  | |  |
|  |  |  | 1 | | 2 | |  | |  |
| Tukey HSD^a^ | Control | 5 | 0.0320 | |  | |  | |  |
|  | B | 5 |  | | 0.5600 | |  | |  |
|  | A | 5 |  | | 0.6140 | |  | |  |
|  | Sig. |  | 1.000 | | 0.053 | |  | |  |
| Means for groups in homogeneous subsets are displayed. | | | | | | |  | |  |
| a. Uses Harmonic Mean Sample Size = 5.000. | | | | | | |  | |  |
|  |  |  |  | |  | |  | |  |
| **Liver** | | | | | | |  | |  |
| Treatment | | N | Subset for alpha = 0.05 | | | |  | |  |
|  |  |  | 1 | | 2 | |  | |  |
| Tukey HSD^a^ | Control | 5 | 0.0740 | |  | |  | |  |
|  | B | 5 |  | | 0.2400 | |  | |  |
|  | A | 5 |  | | 0.2500 | |  | |  |
|  | Sig. |  | 1.000 | | 0.761 | |  | |  |
| Means for groups in homogeneous subsets are displayed. | | | | | | |  | |  |
| a. Uses Harmonic Mean Sample Size = 5.000. | | | | | | |  | |  |
|  |  |  |  | |  | |  | |  |
|  | | | | | | |  | |  |
| **Eye** | | | | | | |  | |  |
| Treatment | | N | Subset for alpha = 0.05 | | | |  | |  |
|  |  |  | 1 | | 2 | | 3 | |  |
| Tukey HSD^a^ | Control | 5 | 0.0660 | |  | |  | |  |
|  | A | 5 |  | | 0.7740 | |  | |  |
|  | B | 5 |  | |  | | 0.8520 | |  |
|  | Sig. |  | 1.000 | | 1.000 | | 1.000 | |  |
| Means for groups in homogeneous subsets are displayed. | | | | | | |  | |  |
| a. Uses Harmonic Mean Sample Size = 5.000. | | | | | | |  | |  |
|  | | | | | | |  | |  |
|  | | | | | | | |  | |
|  | | | | | | | |  |  |
|  | | **Muscle** |  | | | | |  |  |
| Treatment | | N | Subset for alpha = 0.05 | | | | |  |  |
|  |  |  | 1 | 2 | | 3 | |  |  |
| Tukey HSD^a^ | Control | 5 | 0.0640 |  | |  | |  |  |
|  | A | 5 |  | 0.2460 | |  | |  |  |
|  | B | 5 |  |  | | 0.3600 | |  |  |
|  | Sig. |  | 1.000 | 1.000 | | 1.000 | |  |  |
| Means for groups in homogeneous subsets are displayed. | | | | | | | |  |  |
| a. Uses Harmonic Mean Sample Size = 5.000. | | | | | | | |  |  |
|  | | | | | |  | |  |  |
|  | | | | | |  | |  |  |
|  | | **Gonad** |  | | |  | |  |  |
| Treatment | | N | Subset for alpha = 0.05 | | |  | |  |  |
|  |  |  | 1 | 2 | |  | |  |  |
| Tukey HSD^a^ | Control | 5 | 0.0620 |  | |  | |  |  |
|  | B | 5 |  | 0.2520 | |  | |  |  |
|  | A | 5 |  | 0.2560 | |  | |  |  |
|  | Sig. |  | 1.000 | 0.966 | |  | |  |  |
| Means for groups in homogeneous subsets are displayed. | | | | | |  | |  |  |
| a. Uses Harmonic Mean Sample Size = 5.000. | | | | | |  | |  |  |
|  |  |  |  |  | |  | |  |  |
| **Fin** | | | | | |  | |  |  |
| Treatment | | N | Subset for alpha = 0.05 | | |  | |  |  |
|  |  |  | 1 | 2 | |  | |  |  |
| Tukey HSD^a^ | Control | 5 | 0.0320 |  | |  | |  |  |
|  | A | 5 |  | 0.6420 | |  | |  |  |
|  | B | 5 |  | 0.6500 | |  | |  |  |
|  | Sig. |  | 1.000 | 0.861 | |  | |  |  |
| Means for groups in homogeneous subsets are displayed. | | | | | |  | |  |  |
| a. Uses Harmonic Mean Sample Size = 5.000. | | | | | |  | |  |  |

**Table 2.** Concentration of Pb (mg/kg) in gills of *Gambusia affinis*

| Control  (Without PbCl_2_) | 0.1 mg/L PbCl_2_  (A) | 1 mg/L PbCl_2_  (B) |
| --- | --- | --- |
| 0.008 | 0.022 | 0.031 |
| 0.009 | 0.025 | 0.029 |
| 0.007 | 0.021 | 0.03 |
| 0.006 | 0.023 | 0.032 |
| 0.009 | 0.021 | 0.029 |

**Statistical test of Pb levels in gills**

**Normality test**

| **Normality** | | | | | | | |
| --- | --- | --- | --- | --- | --- | --- | --- |
| Treatment | | Kolmogorov-Smirnov^a^ | | | Shapiro-Wilk | | |
|  |  | Statistic | df | Sig. | Statistic | df | Sig. |
| Pb | .00 | 0.221 | 5 | .200^*^ | 0.902 | 5 | 0.421 |
|  | 1.00 | 0.201 | 5 | .200^*^ | 0.881 | 5 | 0.314 |
|  | 2.00 | 0.221 | 5 | .200^*^ | 0.902 | 5 | 0.421 |
| *. This is a lower bound of the true significance. | | | | | | | |
| a. Lilliefors Significance Correction | | | | | | | |

**ANOVA**

| **ANOVA** | | | | | |
| --- | --- | --- | --- | --- | --- |
| Pb | | | | | |
|  | Sum of Squares | df | Mean Square | F | Sig. |
| Between Groups | 0.001 | 2 | 0.001 | 312.806 | 0.000 |
| Within Groups | 0.000 | 12 | 0.000 |  |  |
| Total | 0.001 | 14 |  |  |  |

**Tukey HSD test**

| Treatment | | N | Subset for alpha = 0.05 | | |
| --- | --- | --- | --- | --- | --- |
|  |  |  | 1 | 2 | 3 |
| Tukey HSD^a^ | Control | 5 | 0.0078 |  |  |
|  | A | 5 |  | 0.0224 |  |
|  | B | 5 |  |  | 0.0302 |
|  | Sig. |  | 1.000 | 1.000 | 1.000 |
| Means for groups in homogeneous subsets are displayed. | | | | | |
| a. Uses Harmonic Mean Sample Size = 5.000. | | | | | |

**Table 3.** Concentration of Pb (mg/L) in media (water)

| Control  (Without PbCl_2_) | 0.1 mg/L PbCl_2_  (A) | 1 mg/L PbCl_2_  (B) |
| --- | --- | --- |
| 0.008 | 0.012 | 0.019 |
| 0.006 | 0.013 | 0.02 |
| 0.002 | 0.011 | 0.021 |

**Statistical test** **of Pb levels in treatment media (water)**

**Normality test**

| **Tests of Normality** | | | | | | | |
| --- | --- | --- | --- | --- | --- | --- | --- |
| Treatment | | Kolmogorov-Smirnov^a^ | | | Shapiro-Wilk | | |
|  |  | Statistic | df | Sig. | Statistic | df | Sig. |
| Water | Control | 0.253 | 3 |  | 0.964 | 3 | 0.637 |
|  | A | 0.175 | 3 |  | 1.000 | 3 | 1.000 |
|  | B | 0.175 | 3 |  | 1.000 | 3 | 1.000 |
| a. Lilliefors Significance Correction | | | | | | | |

**ANOVA**

| **ANOVA** | | | | | |
| --- | --- | --- | --- | --- | --- |
| Pb in water | | | | | |
|  | Sum of Squares | df | Mean Square | F | Sig. |
| Between Groups | 0.000 | 2 | 0.000 | 42.824 | 0.000 |
| Within Groups | 0.000 | 6 | 0.000 |  |  |
| Total | 0.000 | 8 |  |  |  |

**Tukey HSD test**

| perlakuan | | N | Subset for alpha = 0.05 | | |
| --- | --- | --- | --- | --- | --- |
|  |  |  | 1 | 2 | 3 |
| Tukey HSD^a^ | Control | 3 | 0.0053 |  |  |
|  | A | 3 |  | 0.0120 |  |
|  | B | 3 |  |  | 0.0200 |
|  | Sig. |  | 1.000 | 1.000 | 1.000 |
| Means for groups in homogeneous subsets are displayed. | | | | | |
| a. Uses Harmonic Mean Sample Size = 3.000. | | | | | |
